# Supplementary material for: Circulating Cancer Associated Macrophage-like Cells as a Potential New Prognostic Marker in Pancreatic Ductal Adenocarcinoma
Source: Biomedicines. 2022 Nov 17;10(11):2955. doi: 10.3390/biomedicines10112955 (PMC9687633; doi:10.3390/biomedicines10112955)
Supplement: Supplementary file 1 [file biomedicines-10-02955-s001.zip › biomedicines-1921878-supplementary.pdf]

Supplementary Table S1: Correlation of CAML detection at follow-up with clinicopathological parameters in curative patients

		CAML				
		none detected at follow-up n=12		≥ 1 CAML detected during follow-up n=10		
		n	%	n	%	p-value
Age	≤ 67 years	5	41.7	7	70.0	
	> 67 years	7	58.3	3	30.0	0.231
Gender	male	3	25.0	6	60.0	
	female	9	75.0	4	40.0	0.192
ECOG	0	4	33.3	9	90.0	
	1	7	58.3	1	10.0	
	2	1	8.3	0	0.0	**0.026**
Neoadjuvant treatment	no	9	75.0	6	60.0	
	yes	3	25.0	4	40.0	0.652
Surgical procedure	PD/PPPD	8	66.7	7	70.0	
	left pancreatectomy	2	16.7	2	20.0	
	total pancreatectomy	2	16.7	1	10.0	0.896
Adjuvant treatment	yes	2	16.7	0	0.0	
	no	10	83.3	10	100.0	0.481
Dindo classification	0-2	8	66.7	4	40.0	
	3-4	4	33.3	6	60.0	0.391
pT stage	T1-2	6	50.0	5	50.0	
	T3-4	6	50.0	5	50.0	1.000
pN stage	N0	5	41.7	1	10.0	
	N+ (N1/2)	7	58.3	9	90.0	0.162
Grading[1]	G2	7	63.4	7	77.8	
	G3	4	36.6	2	22.2	0.642
R status	R0, CRM-	9	75.0	7	70.0	
	R0, CRM+ / R1	3	25.0	3	30.0	1.000

[1] For n=2 patients no grading (G) is available

UICC	I-II	9	75.0	4	40.0	0.192
	III	3	25.0	6	60.0	
Ca 19-9	≤ 500U/ml	9	75.0	8	80.0	1.000
	> 500U/ml	3	25.0	2	20.0	
Recurrence	no	6	50.0	1	10.0	0.074
	yes	6	50.0	9	90.0	
CTC detection at follow-up	no	8	66.7	5	50.0	0.666
	yes	4	33.3	5	50.0	

ECOG, Eastern Cooperative Oncology Group; CRM, circumferential resection margin; Ca 19-9, Carbohydrate Antigen 19-9; UICC, Union for International Cancer Control; PD, partial pancreatoduodenectomy; PPPD, pylorus preserving pancreatoduodenectomy

Supplementary Table S2: Patient characteristic and overall survival curative and palliative cohort including CAML detection

A) OS curative		n=33[§]	Median OS, months (95% CI)	p-value
Age	≤ 67 years	17	21.4 (18.9-20.0)*	0.603
	> 67 years	16	21.2 (15.9-24.4)*	
Gender	male	16	18.4 (14.1-22.7)*	0.030
	female	17	23.1 (21.4-24.8)*	
ECOG	0	20	13.4 (8.0-21.8)*	0.664
	1	12	7.8 (2.3-14.1)*	
	2	33	9.0 (8.0-10.0)*	
UICC stage	I-II	26	22.0 (19.2-24.8)*	0.125
	III	7	14.8 (12.5-17.0)*	
Ca 19-9	<500 U/ml	24	21.2 (18.2-24.2)*	0.991
	≥500 U/ml	9	17.9 (14.9-20.8)*	
R-status	R0; CRM-	17	18.2 (15.6-20.8)*	0.813
	CRM+ /R1	16	21.5 (17.9-25.1)*	
Grading ~	G2	21	20.0 (16.4-23.5)*	0.396
	G3	9	19.8 (17.6-21.9)*	
Neoadjuvant treatment	no	26	20.7 (17.7-23.8)*	0.484
	yes	7	18.5 (17.6-19.4)*	
Adjuvant treatment	no	4	6.8 (0.7-12.8)*	**<0.001**
	yes	29	23 (21.2-24.8)*	
Clavien-Dindo	0-2	19	24.0 (22.1-25.9)*	0.054
	3-4	14	17.4 (15.3-19.6)*	
CAMLs detected at FUP #	no	12	19.6 (6.4-22.8)*	**0.031**
	yes	10	7.0 (2.3-10.1)	

B) OS palliative		n=19	Median OS, month (95% CI)	p-value
Age	≤67 years	9	17.0 (7.7-26.3)	**0.023**
	>67 years	10	3.0 (0.9-5.1)	
Gender	male	14	10.0 (4.6-1.1)	0.709
	female	5	11.8 (3.6-19.9)*	
ECOG	0	5	17.0 (0-35.9)	0.571
	1	11	10.0 (0-20.6)	
	2	3	5.0 (0-11.4)	
UICC stage	III	3	17.0 (0-36.2)	0.745
	IV	16	10.0 (0.9-19.1)	
CAMLs detected at FUP #	no	5	5.0 (0-19.7)	0.220
	yes	6	18.8 (14.9-22.6)*	

A+B) Univariate analyses (p-values, log-rank test).

* median not reached; mean was used; # FUP number of patients § n=3 perioperatively deceased patients not included in the analyses; ~ grading not available for n=3 patients

OS, overall survival; CI, confidence interval; ECOG, Eastern Cooperative Oncology Group; UICC, Union for International Cancer Control; CRM, circumferential resection margin; Ca 19-9, Carbohydrate Antigen 19-9.

Supplementary Table S3. Ca19-9 values and CAML status in the FUP samples of the curative cohort.

Patient	Pre-OP Ca19-9 U/ml	3 month FUP Ca19-9	3 month FUP CAML	6 month FUP Ca19-9	6 month FUP CAML	9 month FUP Ca19-9	9 month FUP CAML	12 +15 month FUP Ca19-9	12 +15 month FUP CAML	FUP CAML pos/neg	INCREASE Ca19-9 at time of CAML positivity	relapse	RFS (months)
**UKE001**	13	8	NEG	18	NEG			21	NEG	NEG	**both neg**	no	25
**UKE021**	4	5	NEG			6	NEG			NEG	**both neg**	no	24
**UKE023**	212					58	NEG			NEG	**n.e.**	no	21
**UKE058**	49			43	NEG			38	NEG	NEG	**both neg**	no	15
**UKE037**	1413	38	POS							POS	**CAML pos.**	no	18
**UKE077**	7209	16710	NEG	79	NEG					NEG	**both neg**	no	16
**UKE092**	37	19	NEG	n.a.	NEG					NEG	**both neg**	no	13
**UKE020**	2			1	NEG	1	POS			POS	**only CAML pos.**	yes	9
**UKE027**	138	9	NEG	11	NEG					NEG	**both neg**	yes	12
**UKE032**	73	10	POS	26	POS					POS	**CAML pos before Ca19-9**	yes	12
**UKE076**	3562			115	NEG	13000	NEG			NEG	**only Ca19-9 pos.**	yes	9
**UKE008**	43	53	NEG	4593	NEG	165	NEG	n.a.	POS	POS	**Ca19-9 before CAML**	yes	6
**UKE028**	19	16	NEG	736	POS	12	POS			POS	**both pos at same time**	yes	5
**UKE036**	125	84	POS	95	POS					POS	**only CAML pos.**	yes	12
**UKE040**	92	264	POS	2010	POS					POS	**both pos at same time**	yes	10
**UKE049**	n.a.			1	NEG	1	NEG			NEG	**both neg**	yes	15
**UKE072**	1380					377	POS			POS	**n.e.**	yes	9
**UKE016**	37			37	NEG	6	NEG			NEG	**both neg**	yes	9
**UKE031**	1474			30	NEG	1334	NEG			NEG	**only Ca19-9 pos.**	yes	12
**UKE056**	47	76	POS	180	NEG	570	NEG			POS	**both pos at same time**	yes	6
**UKE066**	59	647	POS	66	POS					POS	**both pos at same time**	yes	3
**UKE073**	43	20	NEG	24	NEG					NEG	**both neg**	yes	12

n.a. : not evaluable
